# Diagnostic reliability of monitoring for premature atrial and ventricular complexes

**DOI:** 10.1093/europace/euae198

**Published:** 2024-07-26

**Authors:** Alexandra Måneheim, Johan Economou Lundeberg, Anders P Persson, Albin Edegran, Agnieszka Grotek-Cuprjak, Tord Juhlin, Juan Benezet-Mazuecos, Kenneth A Ellenbogen, Gunnar Engström, Jeff S Healey, Linda S Johnson

**Affiliations:** Department of Clinical Sciences, Lund University, Malmö, Sweden; Department of Clinical Sciences, Skåne University Hospital, Carl Bertil Laurells gata 9, 214 28 Malmö, Sweden; Department of Clinical Sciences, Lund University, Malmö, Sweden; Department of Clinical Sciences, Skåne University Hospital, Carl Bertil Laurells gata 9, 214 28 Malmö, Sweden; Department of Clinical Sciences, Lund University, Malmö, Sweden; Department of Clinical Sciences, Skåne University Hospital, Carl Bertil Laurells gata 9, 214 28 Malmö, Sweden; Department of Clinical Sciences, Lund University, Malmö, Sweden; MEDICALgorithmics, Warsaw, Poland; Department of Cardiology, Skåne University Hospital, Malmö, Sweden; Department of Cardiology, Hospital Universitario La Luz, Madrid, Spain; Department of Internal Medicine, Cardiology Division/Pauley Heart Center, Virginia Commonwealth University, Richmond, VA, USA; Department of Clinical Sciences, Lund University, Malmö, Sweden; Population Health Research Institute (PHRI), Hamilton, ON, Canada; Division of Cardiology, McMaster University, Hamilton, ON, Canada; Department of Clinical Sciences, Lund University, Malmö, Sweden; Population Health Research Institute (PHRI), Hamilton, ON, Canada

**Keywords:** Premature ventricular complexes, Premature atrial complex, Ambulatory electrocardiography, Mobile cardiac telemetry, Diagnostic yield

## Abstract

**Aims:**

Short-term ambulatory electrocardiogram (ECG) monitoring is often used to assess premature atrial complex (PAC) and premature ventricular complex (PVC) frequency, but the diagnostic reliability is unknown. The objective of this study was to study the day-to-day variability of PAC and PVC frequency.

**Methods and results:**

We used 14-day full-disclosure mobile cardiac telemetry recordings without atrial fibrillation in 8245 US patients aged 17–103 years to calculate the diagnostic reliability of shorter ambulatory ECG recordings compared with 14-day averages. Over 14 days, 1853 patients had ≥500 PACs/day, 410 patients had ≥5000 PACs/day, and 197 patients had ≥10 000 PACs/day; 1640 patients had ≥500 PVCs/day, 354 patients had ≥5000 PVCs/day, and 175 patients had ≥10 000 PVCs/day. After 3 days, the estimated daily PAC frequency differed by ≥50% from the 14-day mean in 25% of patients; for PVCs, the corresponding duration was 7 days. Ten days of monitoring were needed to estimate PAC and PVC frequency within ±20% of the overall 14-day frequency in 80% of patients. For daily PAC and PVC frequencies ≥10 000, single-day estimation had a specificity of 99.3% [95% confidence interval (CI) 99.1–99.5] at a sensitivity of 76.6 (95% CI 70.1–80.4%) for PACs and a 99.6% (95% CI 99.4–99.7%) specificity at 79.4 (95% CI 72.7–85.2) sensitivity for PVCs. After 7 days, the sensitivity increased to 88.8% (95% CI 83.6–92.9) for PACs and 86.9% (95% CI 80.9–91.5%) for PVCs.

**Conclusion:**

While there is substantial daily variability across most PAC and PVC levels, findings of ≥10 000 PACs or PVCs are highly specific and do not need to be confirmed with longer recordings.

## Graphical Abstract

**Graphical Abstract:**
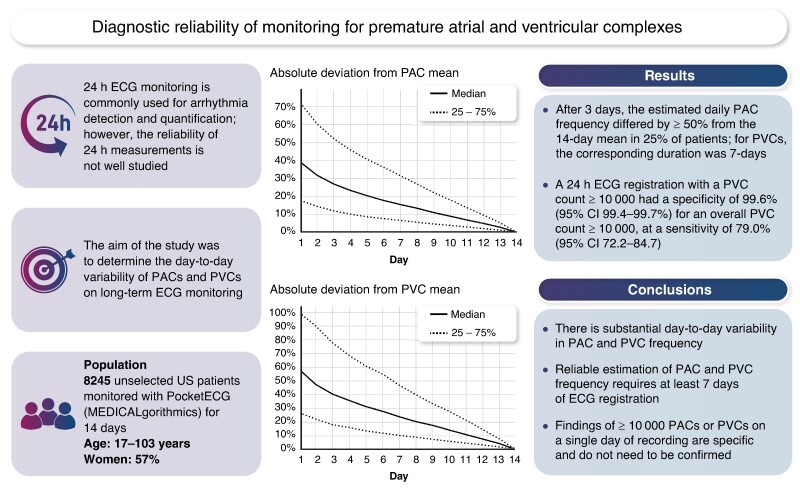

**What’s new?**

- Premature atrial complex (PAC) and premature ventricular complex (PVC) frequencies vary substantially from day to day, irrespective of age or sex. Reliable PAC and PVC quantification requires ≥7 days of recording.
- On single-day recordings, findings of ≥10 000 PACs or PVCs are highly specific, and do not need to be confirmed with longer recordings.

## Introduction

Premature atrial and ventricular beats are both markers of incident disease risk. Premature atrial complexes (PACs) are associated with incident atrial fibrillation (AF) and stroke,^1–8^ and premature ventricular complexes (PVCs) are independently associated with incident heart failure (HF) and AF risk.^9–13^ Among subjects with excessive supraventricular activity, which is linked to stroke independently of clinical AF,^5^ the risk of AF is almost three times higher.^2^ A current consensus document recommends screening for AF in patients with ≥500 PACs/24 h, and referral to a specialist to rule out underlying cardiovascular heart disease inpatients with ≥500 PVCs/24 h.^14^ Besides this role in disease prediction, estimation of PAC and PVC frequency also has clinical relevance, for example, in patients with cardiomyopathy or patients being treated for frequent PVCs, in whom repeated measurements with 24–48 h electrocardiogram (ECG) are often used to assess treatment effect.^15^ The diagnostic reliability of short ambulatory ECG registrations to estimate PAC and PVC frequency is not well studied, however. At the same time, given that there is substantial temporal variability in AF occurrence,^16^ recent developments in digital and remote health make adaptation of monitoring strategies according to initial findings more feasible.^17^

We aimed to determine the day-to-day variability of PACs and PVCs on long-term ECG monitoring in a large unselected patient cohort with mobile cardiac telemetry (MCT) recordings.

## Methods

### Data collection

PocketECG is an FDA- and MDR-certified MCT device that records and transmits a full-disclosure ECG for up to 31 days using limb lead configuration (Leads II and III), and a sampling rate of 300/s. All arrhythmias were detected using an FDA-approved artificial intelligence algorithm and all arrhythmic events were manually verified by certified ECG technicians. Technicians inspected heart rate trends, grouped beats by morphology, and were aided by algorithms that allowed them to filter beats by certain characteristics, for example, tools that detected all beats with pair-interval rates ≥20 b.p.m. faster than the preceding interval. Atrial fibrillation was defined as irregular rhythm without discernible P-waves with a duration ≥30 s. Supraventricular tachycardias and ventricular tachycardias (VTs) were defined as ≥4 consecutive PAC or PVC beats, with a rate ≥100 beats/min. The correlation between PACs and PVCs was calculated using Spearman’s rho.

### Study population

The study sample was derived from a database consisting of all US patients aged 17–103 years who had been referred to ambulatory full-disclosure ECG using the PocketECG device in 2021 with ≥2 days of registration with ≥80% diagnostic signal (*n* = 19 941). We included patients who had ≥14 full days of ECG recordings (*n* = 9677). We excluded patients with AF on Days 1–14 (*n* = 1432), resulting in a population of 8245 patients.

### Statistical methods

Since both the overall mean and the maximal daily PAC and PVC counts could have diagnostic relevance, we analysed PAC and PVC variability in terms of both of these aspects. First, we tabulated 24 h PAC and PVC counts compared with the 14-day overall average across pre-specified strata of 0–99, 100–499, 500–999, 1000–4999, 5000–9999, and ≥10 000, and calculated the sensitivity, specificity, and positive and negative predictive value (NPV) for mean daily PAC/PVC frequencies obtained after 1, 3, and 7 days compared with the 14-day mean daily PAC/PVC frequencies. We also calculated the intra-class coefficient of the day-to-day consistency in PAC and PVC estimation. We then calculated the registration time required to detect maximal 14-day PAC and PVC frequencies within pre-defined levels (500–1999, 2000–4999, 5000–9999, and ≥ 10 000 PVCs), both overall and by strata of sex and age </≥70 years, which is presented visually as the proportion of patients detected over the monitoring duration, and as the time in days until 90% of patients with a peak PAC/PVC frequency within each strata had been diagnosed. We also computed the proportion of patients with a VT episode ≥10 beats, and the median [inter-quartile range (IQR)] monitoring time until such an episode was detected. Finally, we computed the duration of monitoring needed to obtain a mean PAC or PVC count that was within either ±20 or ±40% of the overall 14-day average, overall and stratified by pre-defined levels of PACs and PVCs on the first full day.

All statistical analyses were performed using Stata for Mac v17.0 (StataCorp, College Station, TX, USA), Python 3.8.18 for Mac (Python Software Foundation). The study conforms to the Declaration of Helsinki. All analyses were performed on anonymized data, and for this reason, the ethics review board of Sweden has waived the need for an approval of studies using these data (decision number 2019-03227).

## Results

The median age was 70 years (IQR 60–77, range 17–103), and 57% of subjects were women. Monitoring indications are provided in *Table 1*. The most common indication reported was symptoms (including palpitations, chest pain, and shortness of breath), reported in 32% of subjects. Stroke/transient ischemic attack (TIA) was provided as a monitoring indication in 12.3% of patients and AF/atrial flutter in 18.5% of patients.

**Table 1:** Monitoring indications

Indication	*N*	%
Symptom indication, including palpitation, chest pain, shortness of breath	2599	31.5
Stroke/TIA/amaurosis fugax	998	12.1
Coronary artery disease/angina	14	0.2
Atrioventricular block or bundle branch block	102	1.2
Pre-excitation, Wolf–Parkinson–White syndrome, Long QT-Syndrome	8	0.09
Other unspecified conduction disorder	103	1.3
Paroxysmal tachycardia	566	6.9
Atrial fibrillation or flutter	1520	18.4
Premature atrial or ventricular contractions	111	1.4
Sick sinus syndrome, brady-tachy syndrome	21	0.3
Syncope, pre-syncope, dizziness, lightheadedness	2198	26.7
Missing	5	0.06

### Premature atrial complexes

The population median of the daily PAC count was 77 (IQR 18–397), and 1853 patients (22.5%) had ≥500 PACs, 410 patients (5.0%) had ≥5000 PACs, and 197 patients (2.4%) had ≥10 000 PACs/day. There was a high day-to-day PAC variability; among patients with 100–10 000 PACs on the first day of recording, roughly only one to two out of three patients had an overall 14-day PAC count within the same strata (*Table 2*). The intraclass correlation coefficient (ICC) for individual day-to-day consistency was 0.76 [95% confidence interval (CI) 0.75–0.77], *P* < 0.0001 for PAC frequency. *Table 3* reports the sensitivity, specificity, and NPV and positive predictive value comparing monitoring durations of 1, 3, and 7 days with 14-day average daily PAC frequencies and burdens across different strata. The specificity of PAC counts estimations using single days of recording was good for PAC frequencies >500/day or >0.1%. The sensitivity of single-day recordings was good for PAC frequencies <100 or <0.1%, but otherwise relatively low; for overall daily PAC frequencies >10 000/day, the sensitivity of a single day of recording was 76.6 (70.1–80.4), which increased to 88.8 (95% CI 83.6–92.9) for 7-day registrations.

**Table 2:** Estimation of PAC frequency with 1-day recordings vs. 14-day recordings

Overall 14-day PAC frequency						
PAC frequency Day 1	0–99 (*n* = 4518)	100–499 (*n* = 1874)	500–999 (*n* = 556)	1000–4999 (*n* = 887)	5000–9999 (*n* = 213)	10 000 (*n* = 197)
0–99	4312 (95.4%)	501 (26.7%)	54 (9.7%)	42 (4.7%)	3 (1.4%)	2 (1.0%)
100–499	204 (4.5%)	1187 (63.3%)	189 (34.0%)	86 (9.7%)	5 (2.3%)	4 (2.0%)
500–999	2 (<1%)	136 (7.3%)	185 (33.3%)	117 (13.2%)	4 (1.9%)	1 (0.5%)
1000–4999	0 (0.0%)	50 (2.7%)	121 (21.8%)	564 (63.6%)	58 (27.2%)	7 (3.6%)
5000–9999	0 (0.0%)	0 (0.0%)	5 (0.9%)	65 (7.3%)	103 (48.4%)	32 (16.2%)
≥10 000	0 (0.0%)	0 (0.0%)	2 (0.4%)	13 (1.5%)	40 (18.8%)	151 (76.6%)
Total	100%	100%	100%	100%	100%	100%

PAC, premature atrial complex.

**Table 3:** Diagnostic reliability of PAC and PVC frequencies compared with the overall 14-day average

	24 h recording				72 h recording				7-Day recording			
	Sensitivity	Specificity	PPV	NPV	Sensitivity	Specificity	PPV	NPV	Sensitivity	Specificity	PPV	NPV
**Premature atrial contractions**												
*As daily frequencies*												
<100	95.5 (94.9–96.1)	83.7 (82.4–84.4)	87.6 (86.6–88.5)	94.0 (93.1–94.8)	96.4 (95.8–96.9)	88.9 (87.8–89.8)	95.4 (94.6–96.1)	91.2 (90.4–92.0)	98.0 (97.6–98.4)	93.6 (92.7–94.3)	94.8 (94.1–95.4)	97.5 (97.0–98.0)
100–499	63.3 (61.1–65.5)	92.3 (91.7–93.0)	70.9 (68.6–73.0)	89.5 (88.8–90.3)	72.2 (70.1–74.2)	93.8 (93.2–94.4)	77.5 (75.5–79.5)	92.0 (91.3–92.6)	83.5 (81.7–85.1)	96.4 (95.9–96.8)	87.2 (85.5–88.7)	95.2 (94.6–95.7)
500–999	33.3 (29.4–37.4)	96.6 (96.2–97.0)	41.6 (37.0–46.3)	95.2 (94.7–95.7)	42.6 (38.5–46.9)	96.6 (96.2–97.0)	47.6 (43.1–52.1)	95.9 (95.4–96.3)	59.4 (55.1–63.5)	97.4 (97.1–97.8)	62.6 (58.3–66.8)	97.1 (96.7–97.4)
1000–4999	63.6 (60.3–66.8)	96.8 (96.4–97.2)	70.5 (67.2–73.6)	95.7 (95.2–96.1)	70.2 (67.1–73.2)	97.1 (96.7–97.5)	74.5 (71.4–77.4)	96.4 (96.0–96.8)	81.2 (78.4–83.7)	98.3 (97.9–98.5)	84.9 (82.3–87.2)	97.7 (97.4–98.1)
5000–9999	48.4 (41.5–55.3)	98.7 (98.5–99.9)	50.2 (43.2–57.3)	98.6 (98.4–98.9)	54.0 (47.0–60.8)	98.9 (98.6–99.1)	56.1 (49.0–63.0)	98.8 (98.5–99.0)	73.2 (66.8–79.1)	99.2 (98.9–99.4)	70.0 (63.5–75.9)	99.3 (99.1–99.5)
≥10 000	76.6 (70.1–80.4)	99.3 (99.1–99.5)	73.3 (66.7–79.2)	99.4 (99.2–99.6)	83.2 (77.3–88.2)	99.5 (99.4–99.7)	81.6 (75.5–86.7)	99.6 (99.4–99.7)	88.8 (83.6–92.9)	99.7 (99.6–99.8)	88.8 (83.6–92.9)	99.7 (99.6–99.8)
*As % of total beats*												
0–0.09%	95.3 (94.6–95.9)	85.6 (84.5–86.7)	88.3 (87.3–89.2)	94.1 (93.3–94.9)	98.6 (98.2–98.9)	82.1 (80.9–83.3)	86.3 (85.3–87.2)	98.0 (97.5–98.5)	93.9 (93.1–94.6)	96.2 (95.6–96.8)	96.6 (96.0–97.1)	93.2 (92.4–94.0)
0.1–0.99%	71.9 (70.1–73.6)	92.5 (91.8–93.1)	80.5 (78.8–82.1)	88.4 (87.5–89.2)	70.6 (68.7–72.4)	94.0 (93.4–94.6)	83.6 (81.9–85.9)	88.1 (87.2–88.9)	88.3 (87.0–89.6)	93.9 (93.2–94.5)	86.2 (84.8–87.5)	94.9 (94.3–95.5)
1–9.9%	71.6 (68.9–74.2)	96.9 (96.5–97.3)	79.1 (76.5–81.5)	95.4 (94.9–95.9)	73.1 (70.4–75.6)	98.1 (97.8–98.4)	86.3 (84.0–88.4)	95.7 (95.2–96.2)	87.4 (85.4–89.3)	97.8 (97.4–98.1)	86.6 (84.5–88.5)	97.9 (97.6–98.3)
≥10%	82.6 (76.7–87.5)	99.2 (99.0–99.4)	72.8 (66.6–78.4)	99.6 (99.4–99.7)	65.7 (58.8–72.1)	99.9 (99.8–99.9)	92.5 (87.0–96.2)	99.1 (98.9–99.3)	96.1 (92.5–98.3)	99.3 (99.1–99.4)	77.1 (71.5–82.1)	99.9 (99.8–100)
**Premature ventricular contractions**												
*As daily frequencies*												
<100	96.0 (95.4–96.5)	84.0 (82.6–85.2)	90.9 (90.1–91.6)	92.6 (91.6–93.6)	96.9 (96.3–97.3)	88.6 (87.4–89.7)	93.4 (92.7–94.0)	94.4 (93.5–95.2)	97.8 (97.4–98.2)	93.1 (92.1–94.0)	95.9 (95.4–96.4)	96.2 (95.5–96.9)
100–499	60.1 (57.5–62.6)	93.7 (93.1–94.3)	67.1 (64.5–69.7)	91.7 (91.0–92.3)	70.1 (67.7–72.4)	95.1 (94.6–95.6)	75.5 (73.1–77.7)	93.7 (93.1–94.3)	82.1 (80.0–84.1)	96.6 (96.2–97.0)	83.9 (81.8–85.7)	96.2 (95.7–96.6)
500–999	36.5 (32.3–40.8)	96.9 (96.5–97.3)	43.9 (39.1–48.7)	95.9 (95.4–96.3)	51.2 (46.7–55.6)	97.3 (96.9–97.7)	55.8 (51.1–60.3)	96.8 (96.4–97.2)	65.5 (61.2–69.6)	98.2 (97.9–98.5)	70.8 (66.4–74.8)	97.7 (97.4–98.1)
1000–4999	66.8 (63.4–70.1)	97.4 (97.0–97.8)	72.8 (69.3–76.0)	96.6 (96.2–97.0)	75.4 (72.2–78.4)	98.0 (97.7–98.3)	79.7 (76.6–82.5)	97.5 (97.1–97.8)	84.1 (81.4–86.6)	98.8 (98.5–99.0)	87.8 (85.2–90.0)	98.4 (98.0–98.6)
5000–9999	55.3 (47.7–62.7)	98.7 (98.4–98.9)	48.5 (41.5–55.6)	99.0 (98.8–99.2)	55.3 (47.7–62.7)	99.0 (98.7–99.2)	54.4 (46.9–61.8)	99.0 (98.8–99.2)	75.4 (68.4–81.5)	99.3 (99.1–99.5)	71.4 (64.4–77.8)	99.5 (99.3–99.6)
≥10 000	79.4 (72.7–85.2)	99.6 (99.4–99.7)	80.3 (73.6–86.0)	99.6 (99.4–99.7)	82.3 (75.8–87.6)	99.6 (99.4–99.7)	80.4 (73.9–86.0)	99.6 (99.5–99.7)	86.9 (80.9–91.5)	99.7 (99.6–99.8)	86.4 (80.4–91.1)	99.7 (99.6–99.8)
*As % of total beats*												
0–0.99%	98.8 (98.5–99.1)	80.5 (79.1–81.9)	89.1 (88.3–89.3)	97.7 (97.0–98.2)	98.5 (98.1–98.8)	83.8 (82.4–85.0)	90.7 (89.9–91.5)	97.2 (96.5–97.8)	95.9 (95.4–96.5)	95.4 (94.6–96.1)	97.1 (96.6–97.6)	93.6 (92.7–94.4)
0.1–0.99%	70.9 (68.8–72.9)	94.2 (93.6–94.7)	79.2 (77.2–81.1)	91.2 (90.4–91.8)	73.1 (71.1–75.1)	95.4 (94.8–95.9)	83.2 (81.3–84.9)	91.9 (91.2–92.5)	85.8 (84.1–87.3)	95.9 (95.4–96.4)	86.7 (85.1–88.2)	95.6 (95.0–96.1)
1–9.9%	76.1 (73.3–78.7)	97.7 (97.3–98.8)	81.9 (79.3–84.3)	96.7 (96.3–97.1)	75.4 (72.6–78.0)	98.7 (98.4–98.9)	88.8 (86.5–90.8)	96.6 (96.2–97.0)	88.8 (86.7–90.7)	97.9 (97.6–98.3)	85.7 (83.4–87.8)	98.4 (98.1–98.7)
≥10%	82.3 (76.0–87.6)	99.4 (99.2–99.6)	76.0 (69.4–81.8)	99.6 (99.4–99.7)	71.8 (64.7–78.2)	99.9 (99.8–100)	95.6 (90.6–98.4)	99.4 (99.2–99.5)	97.2 (93.7–99.1)	99.4 (99.2–99.5)	77.2 (71.2–82.5)	99.9 (99.9–100)

NPV, negative predictive value; PPV, positive predictive value.

**Figure 1:**
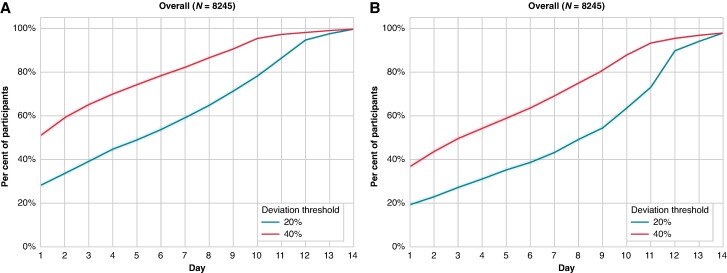
Days needed to achieve mean daily PAC (*A*) or PVC (*B*) frequency within ±20 or ±40% of the overall 14-day average. The shaded area represents a 95% CI.

The day-to-day variability had implications for the monitoring duration needed to accurately estimate PAC frequency. A monitoring duration of over 10 days was needed to estimate the overall 14-day PAC frequency with an error margin of ± 20% in 80% of patients; after 3 days of monitoring, 39.2% of patients had an estimated overall 14-day PAC count within ± 20% of the overall 14-day average (*Figure 1A*). Somewhat shorter monitoring durations were needed to accurately estimate PAC counts among individuals with frequent PACs (Supplementary material online, *Figure S1*).

**Figure 2:**
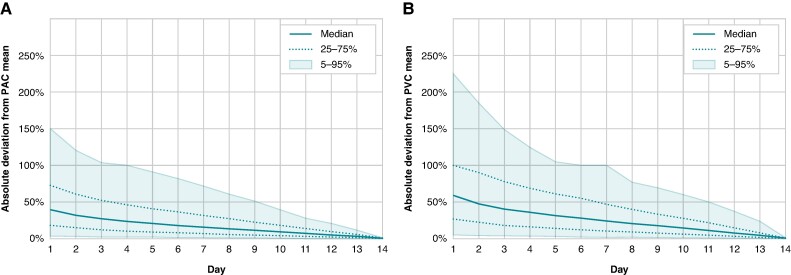
Mean daily PAC (*A*) and PVC (*B*) frequency compared with 14-day average as a function of monitoring time. PAC, premature atrial complex; PVC, premature ventricular complex.

After 4 days of monitoring, the estimated mean daily PAC frequency differed by ≥50% from the mean PAC frequency estimated after 14 days of monitoring in 25% of patients (*Figure 2A*).

**Figure 3:**
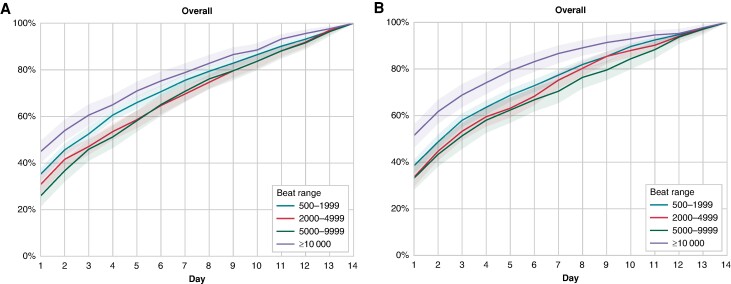
Time to detection of maximal daily PAC (*A*) and PVC (*B*) counts, stratified by maximal daily PAC and PVC counts over 14 days of registration. The shaded area represents 95% CIs.

After 7 days of registration, the maximal PAC count had been detected in 75% of patients with a max of 500–1999, 69.5% of patients with a max of 2000–4999, 70.8% of patients with a max of 5000–9999, and 78.9% of patients with a max of ≥10 000 (*Figure 3A*). Similar results were seen in patients above and below 70 years, and in men and women (Supplementary material online, *Figure S2A–D*).

### Premature ventricular complexes

The population median of the daily PVC count was 32 (IQR 4–294). There were 1640 patients (19.9%) with ≥500 PVCs, 354 patients (4.3%) with ≥5000 PVCs, and 175 patients (2.1%) with ≥10 000 PVCs/day in average. Daily PVC frequencies also varied substantially (*Table 4*). The ICC for consistency between days was 0.87 (95% CI 0.86–0.87), *P* < 0.0001 for PVCs. The sensitivity, specificity, and NPV and positive predictive value for monitoring durations of 1, 3, and 7 days across different strata of PVC frequencies and burdens are reported in *Table 3*. Single-day recordings were highly specific for PVC counts >10 000/day, or >10%, but the sensitivity was limited, at 79.4 (72.7–85.2). Most patients with an overall PVC count ≥ 10 000 who did not have ≥ 10 000 PVCs on the first day of recording had 5000–9999 PVCs on this day, however (*Table 4*).

**Table 4:** Estimation of PVC frequency with 1-day recordings vs. 14-day recordings

Overall 14-day PAC frequency						
PAC frequency Day 1	0–99 (*n* = 5158)	100–499 (*n* = 1448)	500–999 (*n* = 510)	1000–4999 (*n* = 775)	5000–9999 (*n* = 179)	10 000 (*n* = 175)
0–99	4950 (96.0%)	411 (28.4%)	46 (9.0%)	28 (3.6%)	1 (0.6%)	0 (0.0%)
100–499	204 (4.0%)	870 (60.1%)	166 (32.5%)	54 (7.0%)	2 (1.1%)	0 (0.0%)
500–999	4 (0.1%)	131 (9.0%)	186 (36.5%)	96 (12.4%)	6 (3.4%)	1 (0.6%)
1000–4999	0 (0.0%)	36 (2.5%)	109 (21.4%)	518 (66.8%)	42 (23.5%)	7 (4.0%)
5000–9999	0 (0.0%)	0 (0.0%)	3 (0.6%)	74 (9.5%)	99 (55.3%)	28 (16.0%)
≥10 000	0 (0.0%)	0 (0.0%)	0 (0.0%)	5 (0.6%)	29 (16.2%)	139 (79.4%)
Total	100%	100%	100%	100%	100%	100%

PAC, premature atrial complex.

After 7 days of monitoring, the overall 14-day average PVC count still deviated by ≥50% in 25% of the population (*Figure 2B*). Monitoring durations of more than 11 days were needed to estimate the overall 14-day PVC frequency with an error margin of ±20% in 80% of patients (*Figure 1B*). After 3 days of monitoring, <30% of patients had an estimated daily PVC count within ±20% of the overall 14-day average (*Figure 1B*). Somewhat shorter monitoring durations were needed in patients with more frequent PVCs on the first day of registration (Supplementary material online, *Figure S3A–D*). After 1 week of monitoring, maximal PVC counts had been detected in 77.2% of patients with a maximal daily PVC counts of 500–1999, 75.0% patients with a maximal PVC count of 2000–4999, 70.3% of patients with a maximal PVC count of 5000–9999, and 86.3% of patients with a maximal PVC count of ≥10 000 (*Figure 3B*). Similar results were found in both men and women, and patients aged above or below 70 years (Supplementary material online, *Figure S4A–D*). Ventricular tachycardia episodes ≥10 beats were detected in 922 patients (11.2%) after a median recording duration of 6 (IQR 3–10) days.

## Discussion

In this unselected sample of adult patients undergoing ≥14 days of full-disclosure ambulatory ECG, there was a pronounced temporal variability of PAC and PVCs; after 10 days of monitoring, the estimated PAC or PVC frequency still differed by more than 20% compared with the 14-day average in one-fifth of the population, and 7 days of monitoring were needed to detect the maximal daily 14-day PAC and PVC frequencies in 75% of patients. Patients with low (<100/day) PAC and PVC frequencies, one in every two patients, could be reliably identified by 1–3 day recordings. Short recordings had good specificity for PAC or PVC frequencies > 10 000 daily, and thus, these findings on 1–3-day recordings do not need to be confirmed.

It is not known whether maximal daily PAC/PVC frequency or mean daily PAC/PVC frequency is the more relevant measure in terms of incident outcome prediction, but ectopic beat frequency is commonly assessed to determine prognosis and inform treatment decisions.^15,18^ Repeated ambulatory ECG examinations are recommended in patients with hypertrophic cardiomyopathy, to detect atrial fibrillation and assess the occurrence of non-sustained VT episodes.^19,20^ Premature ventricular complex frequency is also estimated in patients at risk of arrhythmia-induced cardiomyopathy,^21–24^ mortality risk,^25,26^ or in whom repeated examinations may be needed to assess the effect of medical treatment, catheter ablation.^23^ Unfortunately, we have no detailed information on clinical characteristics, and therefore, this study cannot directly inform management in these patient populations. Our results, which are supported by previous findings in smaller studies of day-to-day variability among patients with frequent PVCs (>1 or 5%),^27–29^ indicate a need to further study the diagnostic reliability of PACs, PVCs, and VT in specific patient groups. They are also in line with previous studies that demonstrate the need for longer registrations in patients with syncope and palpitations.^30,31^

Premature atrial complexes and PVCs are highly prevalent in the general population.^32–34^ Even modestly raised PAC frequencies^1,2^ are associated with concurrent and incident atrial fibrillation,^35^ while low levels of PVCs predict the risk of HF.^10^ In the light of this, current consensus documents suggest imaging, electrical, and genetic evaluation in patients with ≥500 PVCs/24 h to exclude underlying disease, and screening for AF and sleep apnoea in patients with ≥500 PACs.^14^ We observed such substantial day-to-day variability at PAC and PVC levels of 100–10 000/day that we would recommend 7-day recordings for patients with 100–10 000 beats on an initial 24 h ECG, in patients in whom the PAC or PVC frequency would influence a clinical decision to undertake further examinations.

### Strengths and limitations

The main strength of our study is the use of a large unselected patient cohort with full-disclosure ECG registration and beat-to-beat annotations of all arrhythmic events that has allowed us to report temporal variability, and the influence this has on diagnostic reliability on both PACs and PVCs at all levels. The large patient cohort has also allowed us to report data separately by age and sex strata. The data were downloaded directly from the device manager, and include all patients examined with PocketECG in the USA for a calendar year, which implies generalizability to US patients who undergo MCT monitoring.

One limitation to the study is that the 14-day average daily PAC and PVC frequency, which we used as our gold standard, is an arbitrary measurement. Furthermore, we did not have access to other clinical characteristics which would have been of interest, such as prior HF, the presence of cardiomyopathies, including arrhythmogenic right ventricular cardiomyopathy, long-QT syndrome, or the use of antiarrhythmic drugs, nor did we have access to follow-up data for incident disease diagnoses. Future studies that address the impact of temporal variability of PACs and PVCs on clinical outcome prediction would be of value.

## Conclusions

There is substantial day-to-day variability in PACs and PVCs frequencies, particularly among patients with 100–10 000 PACs/PVCs daily. In patients with ≥10 000 PVCs, a 24 h measurement has an 80% sensitivity and a 99.6% specificity compared with a 14-day mean daily PVC estimation.

## Supplementary Material

euae198_Supplementary_Data

## Data Availability

Data will be provided upon reasonable request to the senior author at linda.johnson@med.lu.se.
