# Supplementary material for: Diagnostic reliability of monitoring for premature atrial and ventricular complexes
Source: Europace. 2024 Jul 26;26(8):euae198. doi: 10.1093/europace/euae198 (PMC11297493; doi:10.1093/europace/euae198)
Supplement: euae198_Supplementary_Data [file euae198_supplementary_data.docx]

DATA SUPPLEMENTS

**Figure S1 Days needed to estimate a mean daily PAC frequency within ±20 or ±40% of the 14-day average, stratified by levels of PACs on the initial 24hECG**

**Figure S2a-d**

**Time to detection of maximal daily PAC counts, stratified by maximal daily PAC and counts over 14 days of registration across age and sex groups**

**Figure S3a-d Days needed to estimate a mean daily PVC frequency within ±20 or ±40% of the 14-day average, stratified by levels of PVCs on the initial 24hECG**

**Figure S4a-d**

**Time to detection of maximal daily PVC counts, stratified by maximal daily PVC and counts over 14 days of registration across age and sex groups**
